# Circulating histone signature of human lean metabolic-associated fatty liver disease (MAFLD)

**DOI:** 10.1186/s13148-020-00917-2

**Published:** 2020-08-20

**Authors:** Diana Buzova, Andrea Maugeri, Antonio Liguori, Cecilia Napodano, Oriana Lo Re, Jude Oben, Anna Alisi, Antonio Gasbarrini, Antonio Grieco, Jan Cerveny, Luca Miele, Manlio Vinciguerra

**Affiliations:** 1 Department of Adaptive Biotechnologies, Global Change Research Institute CAS, Brno, Czech Republic; 2 International Clinical Research Center, St Anne’s University Hospital, Brno, Czech Republic; 3 Department of Medical and Surgical Sciences and Advanced Technologies “GF Ingrassia”, University of Catania, Catania, Italy; 4 Department of Gastroenterological, Endocrine-Metabolic and Nephro-Urological Sciences, Fondazione Policlinico Universitario A. Gemelli IRCCS, Rome, Italy; 5 Institute for Liver and Digestive Health, Division of Medicine, University College London (UCL), London, UK; 6 Research Unit of Molecular Genetics of Complex Phenotypes, Bambino Gesù Children’s Hospital, IRCCS, Rome, Italy

**Keywords:** Liquid biopsy, Histones, Epigenetics, ImageStream, Metabolic health, Lean MAFLD

## Abstract

**Background:**

Although metabolic associate fatty liver disease (MAFLD) is associated with obesity, it can also occur in lean patients. MAFLD is more aggressive in lean patients compared to obese patients, with a higher risk of mortality. Specific biomarkers to diagnose differentially lean or overweight MAFLD are missing. Histones and nucleosomes are released in the bloodstream upon cell death. Here, we propose a new, fast, imaging and epigenetics based approach to investigate the severity of steatosis in lean MAFLD patients.

**Results:**

A total of 53 non-obese patients with histologically confirmed diagnosis of MAFLD were recruited. Twenty patients displayed steatosis grade 1 (0–33%), 24 patients with steatosis grade 2 (34–66%) and 9 patients with steatosis grade 3 (67–100%). The levels of circulating nucleosomes were assayed using enzyme-linked immunosorbent assay, while individual histones or histone dimers were assayed in serum samples by means of a new advanced flow cytometry ImageStream(X)-adapted method. Circulating nucleosome levels associated poorly with MAFLD in the absence of obesity. We implemented successfully a multi-channel flow methodology on ImageStream(X), to image single histone staining (H2A, H2B, H3, H4, macroH2A1.1 and macroH2A1.2). We report here a significant depletion of the levels of histone variants macroH2A1.1 and macroH2A1.2 in the serum of lean MAFLD patients, either individually or in complex with H2B.

**Conclusions:**

In summary, we identified a new circulating histone signature able to discriminate the severity of steatosis in individuals with lean MAFLD, using a rapid and non-invasive ImageStream(X)-based imaging technology.

## Background

Non-alcoholic fatty liver disease (NAFLD) is the most common type of progressive chronic liver disease that consists in accumulation of fat, followed by liver inflammation (non-alcoholic steatohepatitis, NASH) and occurs mostly in patients who are obese and have the metabolic syndrome [1]. Recently, the term NAFLD was re-defined as metabolic (dysfunction)-associated fatty liver disease, or “MAFLD”, a more appropriate nomenclature encompassing clinical features [2]. MAFLD is a leading etiology underlying many cases of hepatocellular carcinoma (HCC), a devastating malignancy [3–5]. Obesity-associated MAFLD affects about 1/3 of the general population in the Western world [6]. Although MAFLD is mostly correlated to obese patients, it can also occur in lean patients: in Asian populations, 27% of lean individuals with normal BMI present with MAFLD, which is unrelated to metabolic syndrome compared to people with high BMI (19% Vs. 61%) [7]; while in Europe and in the USA, a lower prevalence of lean MAFLD/NASH, in this case, associated to the metabolic syndrome, was reported [7]. In lean patients, MAFLD is related to visceral fat accumulation [7]. Lean MAFLD pathogenesis seems to be due, at least in part, to altered gut microbiota composition [8]. Also, genetic predisposition can increase the probability to have MAFLD in lean patients [7]. MAFLD is considered more aggressive in lean patients compared to obese patients for liver disease progression, as assessed histologically [9] and longitudinally [10], with a higher risk of mortality [11]. Other studies challenged this view [12, 13]. So far, specific biomarkers to diagnose differentially lean MAFLD are missing. In an effort to identify new epigenetic liquid biopsies for MAFLD, we have recently shown a strong correlation between fatty liver index [FLI, a simple MAFLD predictor based on BMI, waist circumference, triglycerides and GGT [14]], and high level of circulating nucleosomes in obese patients with metabolic syndrome with MAFLD [15]. Nucleosomes, the basic repeating units of chromatin, allow genome compaction in the cell nuclei, and their composition and post-translational modifications regulate gene expression [16]. Interestingly, intact nucleosome levels in the circulation are elevated in several cancers and in acute conditions such as stroke, trauma and sepsis [17, 18]. Furthermore, a strong diagnostic and prognostic performance for circulating nucleosomes has been reported for pancreatic [19], lung [20], colorectal [21] and breast cancers [22]. The potential of circulating nucleosomes to serve as biomarkers, or “liquid biopsies” is, therefore, a promising area of research for early cancer detection and monitoring treatment responses.

It is unknown if circulating nucleosomes are reliable markers for lean MAFLD. In addition, the variability in histone composition within nucleosomes renders their potential in diagnosis and prognosis vast and promising. Beyond intact nucleosomes, several studies have demonstrated that extracellular histones H3 and/or H4 are potential mediators of lethal systemic inflammatory diseases [23] and inflammation [24]. Moreover, in addition to the “canonical” histones, there exist 19 variants of H2A and 6 variants of H3, in humans [25]. Histone variants differ in their unique temporal pattern of chromatin deposition during the cell cycle [25]. The members of the macroH2A group of H2A histone variants (macroH2A1 and macroH2A2) are the largest in nature [25]. Others and we have recently demonstrated that macroH2A1 isoforms play fundamental roles in modulating stem cell differentiation, MAFLD and HCC progression [26–31]. It is unknown whether individual diverse circulating histone complexes may be used as biomarkers for lean MAFLD. Simultaneous assaying of multiple circulating histones remains very challenging. However, high-resolution imaging, based on ImageStream(X) imaging flow cytometer, could allow quantitative detection of expression of multiple biomarkers, such as histones, on circulating blood and cancer cells, with high reliability-, speed- and low-associated costs [32]. In this study, we point to identify a histone-based signature that is robustly related with the severity of human lean MAFLD by using for the first time an ImageStream(X)-based method to detect circulating histones.

## Results

### Poor association of serum nucleosome levels and non-obese patients with MAFLD

**Fig. 1:**
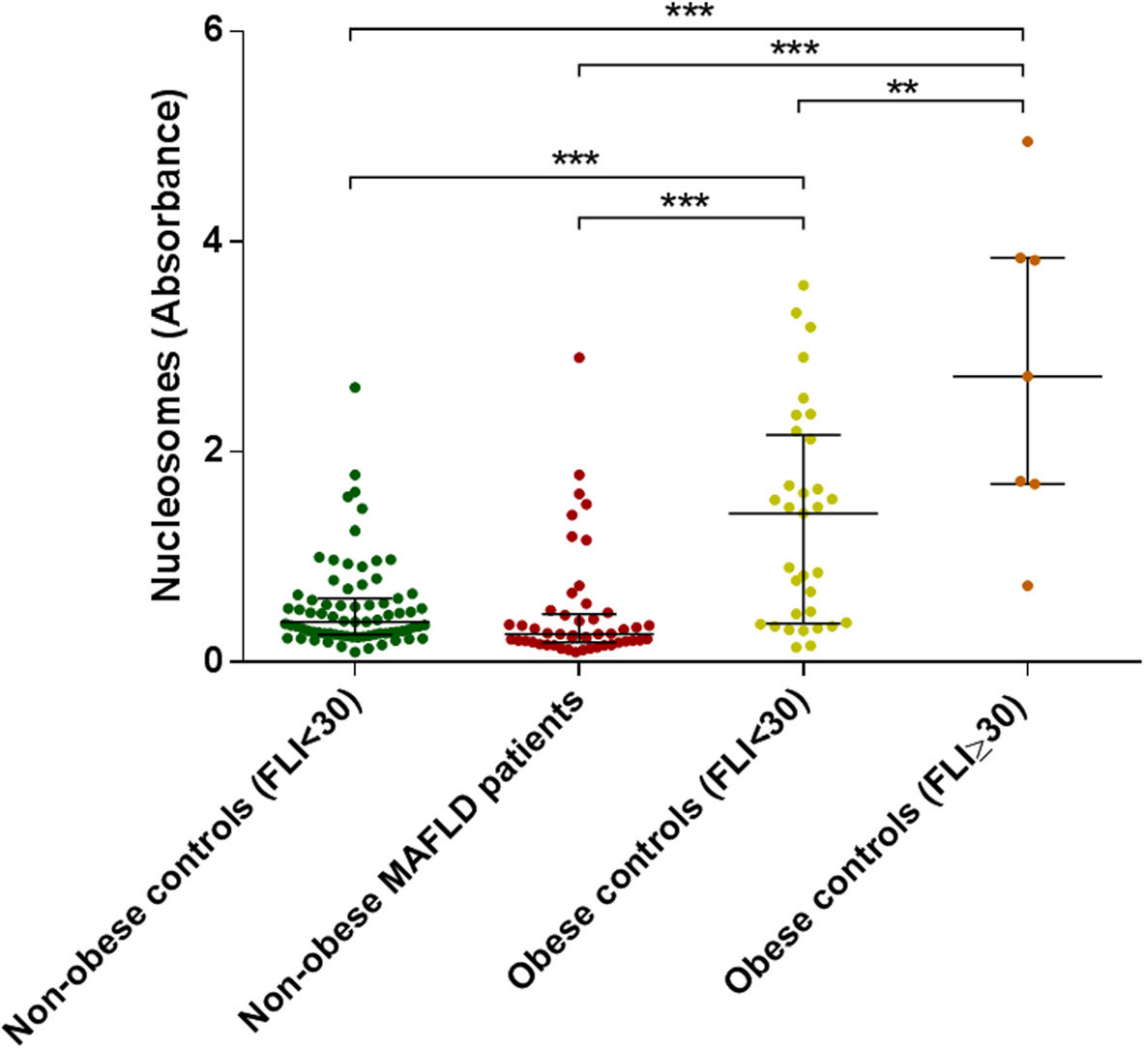
Differences in circulating nucleosome levels between non-obese MAFLD patients (*n* = 53), non-obese controls with fatty liver index (FLI) < 30 (*n* = 80), and obese controls with FLI < 30 or ≥ 30 (*n* = 33 and *n* = 7, respectively). **p < 0.01 and ***p < 0.001 based on the Mann-Whitney *U* test

**Fig. 2:**
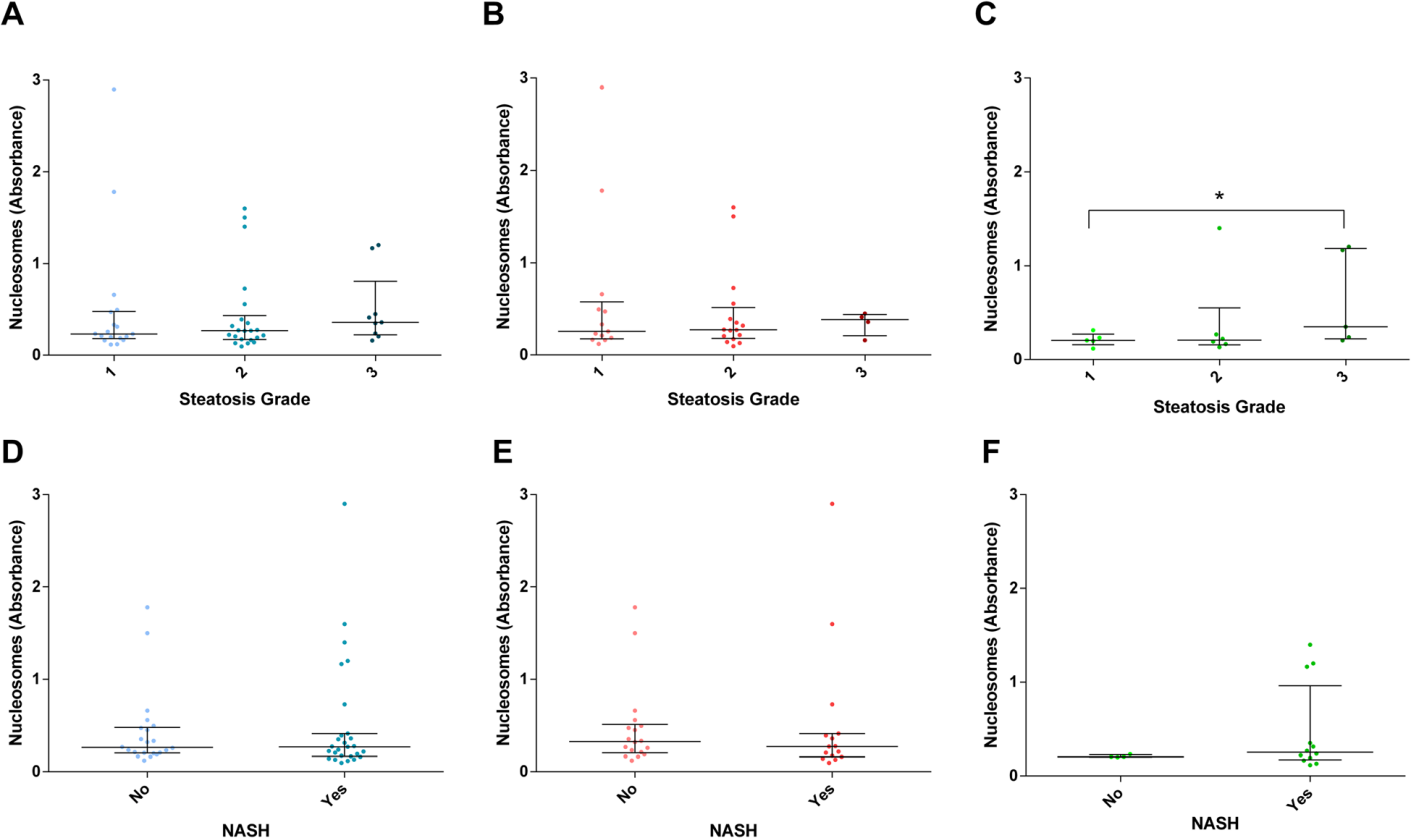
Differences in circulating nucleosome levels according to steatosis grades (1 to 3) and presence of NASH in lean/overweight MAFLD patients (**a**, **d**), *n* = 53; overweight MAFLD patients (**b**, **e**), *n* = 38; lean MAFLD, patients (**c**, **f**), *n* = 15. *p < 0.05 based on the Mann-Whitney *U* test

We have recently demonstrated that circulating nucleosome levels associate strongly with obesity-induced MAFLD, as determined by ELISA assay [15]. Here, we sought to determine if circulating nucleosome levels associate with MAFLD among non-obese patients. To this purpose, a total of 53 lean or overweight patients with biopsy-proven MAFLD were recruited from the outpatient clinic [33] and compared with non-obese or obese individuals with FLI < 30 (*n* = 80 and *n* = 33, respectively) and obese individuals with FLI ≥ 30. As previously demonstrated [15], circulating nucleosome levels were associated with obesity in general. Specifically, non-obese MAFLD patients had lower circulating nucleosome levels than their obese counterparts, while no difference with non-obese controls was evident. We also observed that circulating nucleosome levels were associated with FLI among obese but not among non-obese subjects (Fig. 1). Next, non-obese patients with MAFLD were stratified according to their steatosis grade: 21 patients presented with steatosis grade 1 (S1, 0–33%), 24 patients presented with steatosis grade 2 (S2, 34–66%) and 9 patients presented with steatosis grade 3 (S3, 67–100%). Demographics and characteristics of patients according to steatosis grades are listed in Table 1. Median BMI for all 53 lean or overweight patients was 26.8 (IQR = 24.6–28.7), 23.4 (IQR = 23.0–24.8) for lean patients (*n* = 15) and 28.0 (IQR = 26.3–29.0) for overweight patients (*n* = 38). There were no significant differences in laboratory parameters among patients groups S1-S2-S3 (Table 1). All patients exhibited also, to a variable extent, other histological markers of MAFLD/NASH such as ballooning, lobular inflammation and fibrosis (Table 2). 13/53 patients exhibited a high (> 4) NAS score, representing the sum of scores for steatosis, lobular inflammation and ballooning, and ranging from 0–8 [33]) (Table 2). Surprisingly, we failed to find an association between circulating nucleosome levels and steatosis grade, S1 to S3, in non-obese (lean+overweight) patients (Fig. 2a) and in overweight patients (Fig. 2b), while in lean patients a significant increase (*p* = 0.018) was detected in S3 vs S1 individuals (Fig. 2c). Similarly, no difference in circulating nucleosome levels according to NASH diagnosis was evident (Fig. 2d–f). Overall, these data imply that serum nucleosome levels are not universal markers of MAFLD and NASH in the absence of obesity.

**Table 1:** Characteristics of the study population, by steatosis grade

Variable	All patients (53)	S1 (21)	S2 (24)	S3 (8)	***p*** value *
Male Sex, *n (%)*	44 (83.0)	20 (95.2)	17 (70.8)	7 (87.5)	0.08
Age, *median (IQ range)*	51 (43–55)	49 (40–54)	49 (43–57)	52 (44–54)	0.7
BMI, *median (IQ range)*	26.8 (24.6–28.7)	27.1 (24.9–28.7)	26.1 (24.6–28.7)	26.2 (23.8–29.0)	0.9
Diabetes, *n (%)*	7 (13.2)	2 (9.5)	5 (20.8)	0 (0)	0.2
Dyslipidemia, *n (%)*	27 (50.9)	10 (47.6)	14 (58.3)	3 (37.5)	0.5
Hypertension, *n (%)*	14 (26.4)	3 (14.3)	9 (37.5)	2 (25.0)	0.2
Platelets (×10^9^/ml), *median (IQ range)*	223 (190–270)	228 (161–261)	222 (186–282)	217 (204–286)	0.5
Albumin (g/l), *median (IQ range)*	46 (44–48)	46 (44–48)	48 (46–50)	45 (44–47)	0.2
AST (IU/l), *median (IQ range)*	34 (28–51)	29 (25–50)	32 (27–56)	38 (34–51)	0.4
ALT (IU/l), *median (IQ range)*	60 (50–83)	54 (44–60)	69 (51–89)	80 (60–114)	0.07
g-GT (IU/l), *median (IQ range)*	51 (32–99)	72 (39–124)	50.5 (33–81)	30 (26–63)	0.06
Alkaline phosphatase (IU/l), *median (IQ range)*	118 (70–189)	90 (81–181)	113 (65–187)	182 (113–199)	0.5
Glycemia (mg/dl), *median (IQ range)*	88 (84–96)	85 (81–94)	89.5 (85–98)	88.5 (85–91)	0.3
Fasted insulin (mUI/l), *median (IQ range)*	12.6 (10.1–16.2)	10.6 (8.1–16.2)	12.2 (10.8–15.0)	14 (12.6–27.4)	0.1
HOMA-IR, *median (IQ range)*	2.68 (2.18–3.73)	2.4 (2.0–3.5)	2.7 (2.25–3.70)	3.14 (2.64–6.15)	0.1
Total cholesterol, *median (IQ range)*	203 (178–229)	207 (180–229)	203 (187–245)	179 (170–208)	0.3
HDL (mg/dl), *median (IQ range)*	45 (40–51)	48 (39–55)	45 (42–49)	46 (43–51)	0.7
LDL (mg/dl), *median (IQ range)*	136 (107–152)	136 (107–156)	143 (129–151)	101 (86–106)	0.09
Triglycerides (mg/dl), *median (IQ range)*	126 (85–175)	110 (77–147)	154 (97–197)	135 (108–167)	0.1

*p* value for trend (Kruskal-Wallis test or chi^2^)

**Table 2:** NAFLD/NASH histological scores of the study population (*n*(%))

Variable	All patients (53)
Steatosis	
0	0 (0)
1	21 (39.6)
2	24 (45.3)
3	8 (15.1)
Ballooning	
0	9 (17.0)
1	38 (71.7)
2	6 (11.3)
Inflammation	
0	4 (7.5)
1	40 (75.5)
2	9 (17.0)
3	0 (0)
NAS > 4	13 (24.5)
Fibrosis	
0	2 (3.8)
1	38 (71.7)
2	13 (24.5)
3	0 (0)
4	0 (0)

### Optimization of ImageStream(X) for the detection of circulating histone and histone complexes

**Fig. 3:**
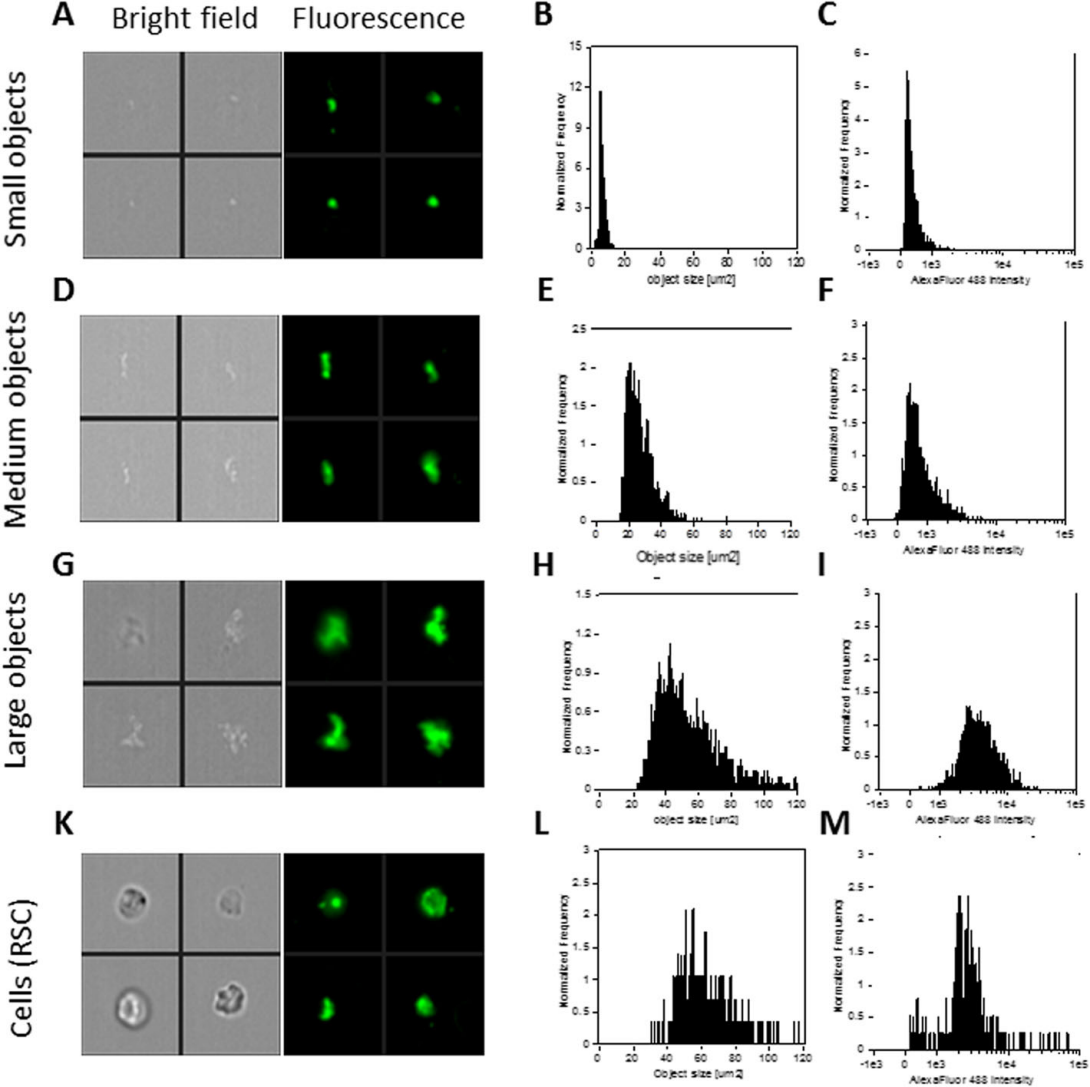
Representative images, size distribution and fluorescence signal intensity from Alexa Fluor® 488 of three types of circulating objects (**a**–**i**) and cells (**k**–**m**) positive for H2A expression. ImageStream photographs show bright-field images and H2A staining (fluorescence from Alexa Fluor® 488)

Given the lack of diagnostic value of circulating nucleosomes for non-obese MAFLD, we sought to determine if, conversely, the diversity of circulating single histones (H2A, H2B, H3, H4, macroH2A1.1 and macroH2A1.2), and histone complexes [23–31] may be used as new biomarkers for lean MAFLD. ELISA assays can detect nucleosome or individual histones (one by one) in serum; however, a real-time high throughput assaying of multiple histones remains unexplored. Here, we developed a multi-channel flow imaging methodology based on ImageStream(X), a device that can routinely detect the expression of multiple biomarkers on circulating cancer cells, with high reliability [32]. First, we optimized the protocol by staining a healthy volunteer blood sample with a single anti-H2A antibody, followed by a secondary antibody Alexa Fluor® 488 (green staining) which was detected by an appropriate channel. 10,000 objects were imaged in a volume of 0.6 μl within 1 min, in bright field and in fluorescence. Gating was applied to discriminate: (a) focused objects, (b) objects with fluorescence, and to exclude round single objects (RSC), corresponding to the cellular fraction. Representative images are shown in Fig. 3. Three overlapping types of circulating objects positive for H2A expression were identified on the basis of object size and fluorescence intensity: a high-frequency population of small size objects (peaking at 0–10 μm^2^) (Fig. 3a–c), a mid-frequency population of medium size objects (peaking at 20–30 μm^2^) (Fig. 3d–f), and a low-frequency population of large-sized objects (peaking at 40–60 μm^2^) (Fig. 3g–i). Interestingly, these object populations together outnumbered RSC, the cell population which accounted only for < 1% of the overall object count. H2A-stained cell size ranged from 40 to 100 μm^2^ (Fig. 3k–m).

**Fig. 4:**
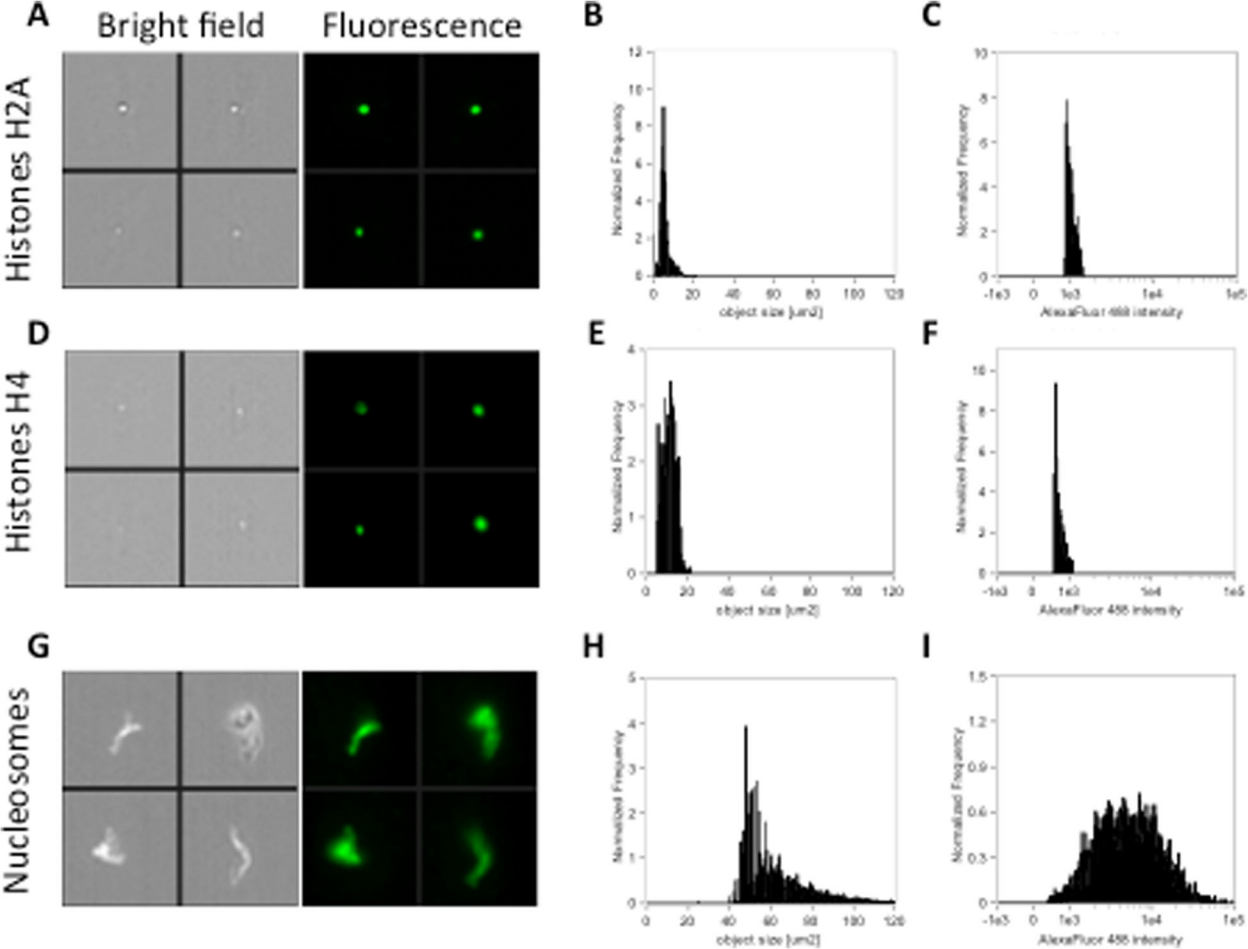
Representative images, size distribution and fluorescence signal intensity from Alexa Fluor® 488 of recombinant core histones H2A (**a**–**c**), recombinant core histones H4 (**d**–**f**) and human native nucleosomes protein (**g**–**i**) positive for H2A (H4) expression. ImageStream photographs show bright-field images and H2A (H4) staining (fluorescence from Alexa Fluor® 488)

Next, we attempted to identify these acellular complexes as true histones/histone complexes, by using as positive controls recombinant core human histones (H2A, H4) or human native nucleosomes, commercially available. Recombinant core histones H2A or H4 solution (20 μg/ml) were imaged with respective specific antibodies in a volume of 0.5 μl within 1 min, in bright field and in fluorescence using the same gating strategy as in Fig. 1. Both histone H2A (Fig. 4a–c) and histone H4 (Fig. 4d,e) were extremely similar in terms of object size to the population of small size objects (Fig. 3a–c), with a tendency towards a more intense fluorescence signal. In a separate experiment, human native nucleosomes protein (100 μg/ml) were incubated with anti-H2A antibody in a volume of 0.36 μl within 1 min, and then imaged in bright field and in fluorescence: H2A-stained native nucleosomes (Fig. 4g–i) in turn appeared very similar, in terms of object size and fluorescence intensity distributions, to the population of large-sized objects (Fig. 3g–i). While we can hypothesize that the population of medium size objects might represent sub-nucleosomal histone complexes, such as dimers, overall, these findings suggest the feasibility of a new robust and high throughput detection methods of histones and histone complexes in human serum.

### Circulating macroH2A1.2, H2B and H4 histones mark the severity of the human lean non-alcoholic fatty liver disease

**Fig. 5:**
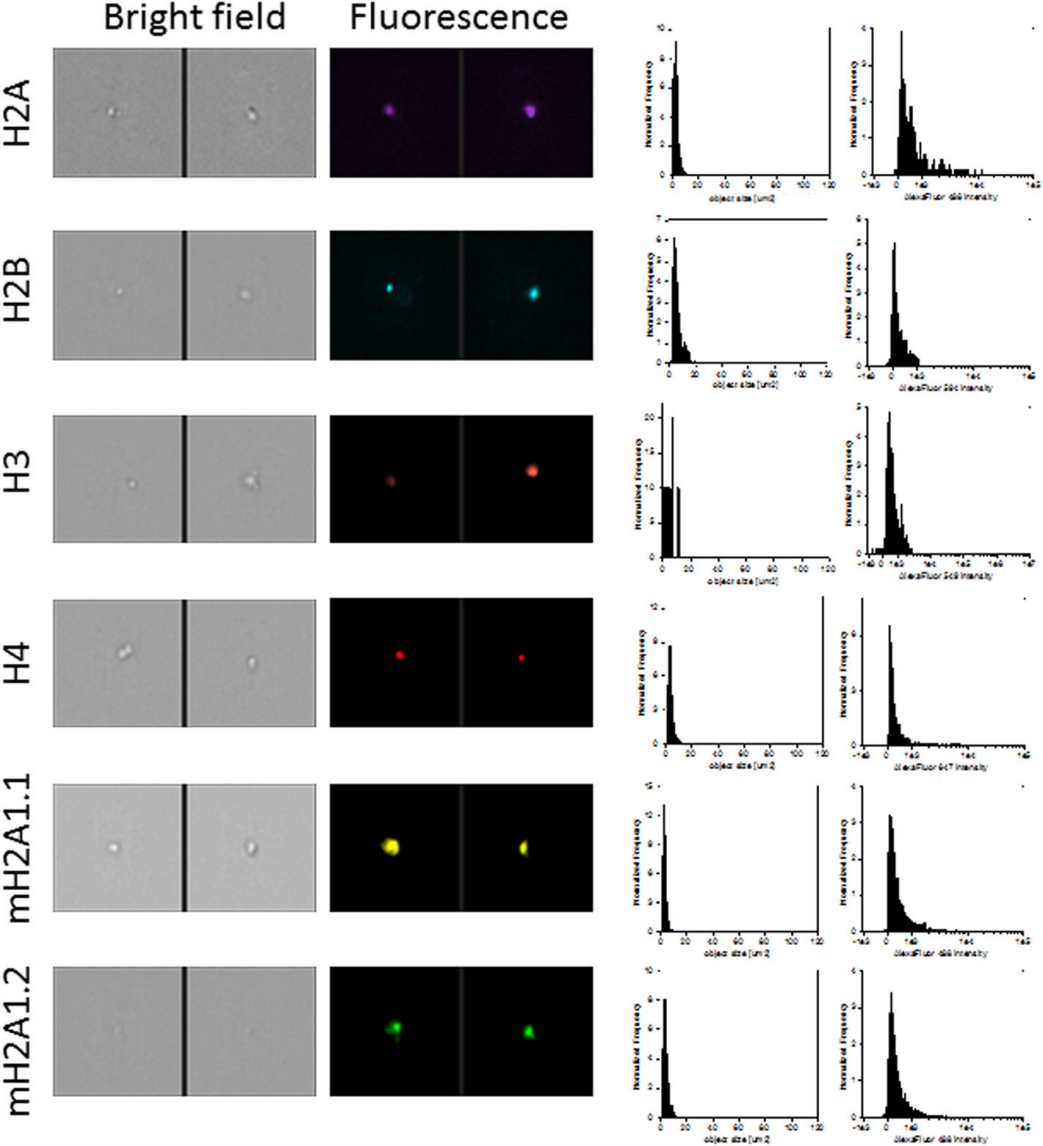
Representative images, size distribution and fluorescence signal intensity from fluorescence marker of canonical histones (H2A, H2B, H3, H4) and 2 large variants of histone H2A (macroH2A1.1 and macroH2A1.2). ImageStream photographs show bright-field images and histone staining (fluorescence from Alexa Fluor® 488, Alexa Fluor® 549, Alexa Fluor® 594 or Alexa Fluor® 647)

**Fig. 6:**
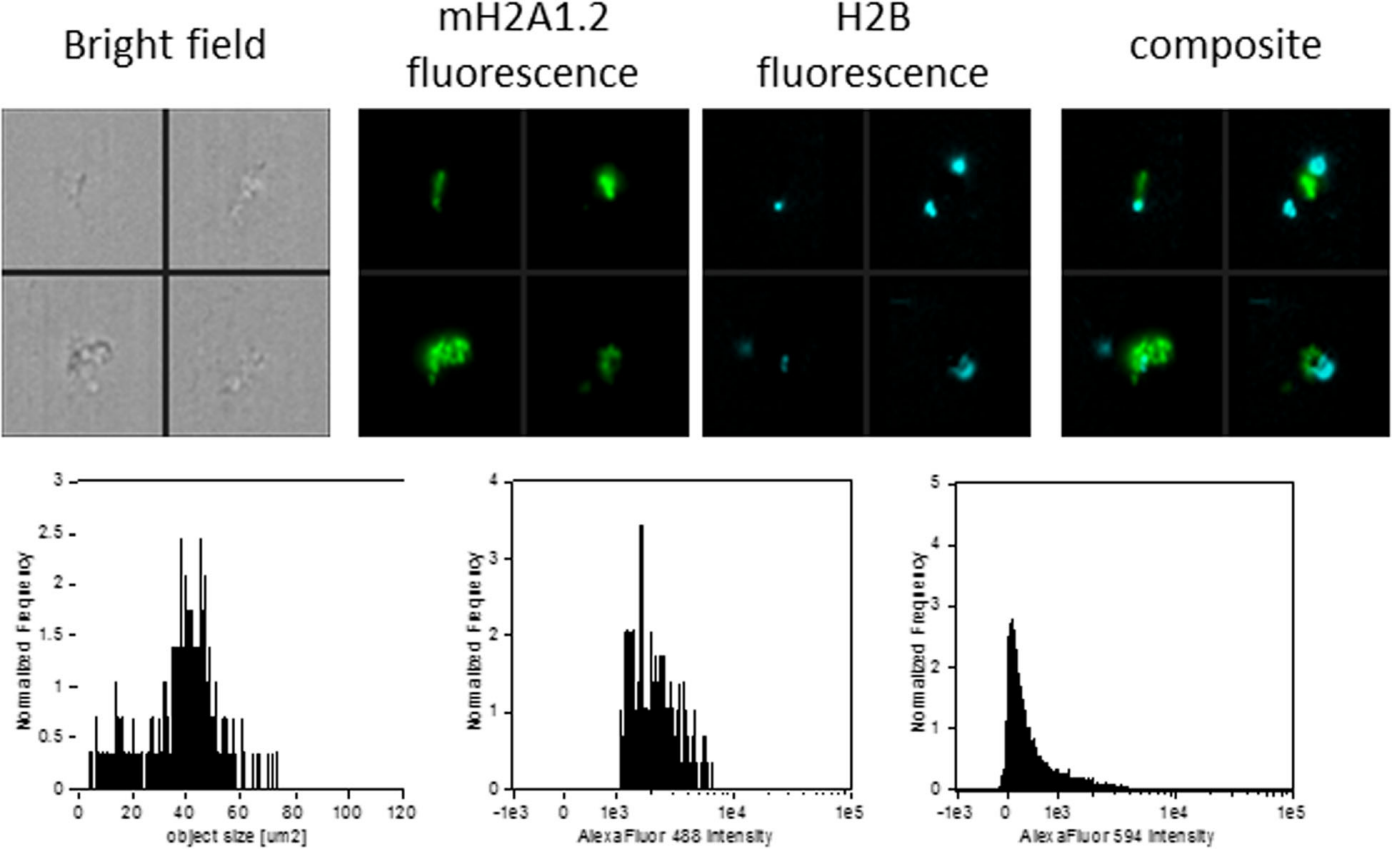
Representative images, size distribution and fluorescence signal intensity from Alexa Fluor® 488 and Alexa Fluor® 594 of multi-channel detecting of macroH2A1.2/H2B histone. ImageStream photographs show bright-field images, macroH2A1.2 histone staining (fluorescence from Alexa Fluor® 488), H2B histone staining (fluorescence from Alexa Fluor® 594) and composite images, overlays of macroH2A1.2 and H2B staining.

**Fig. 7:**
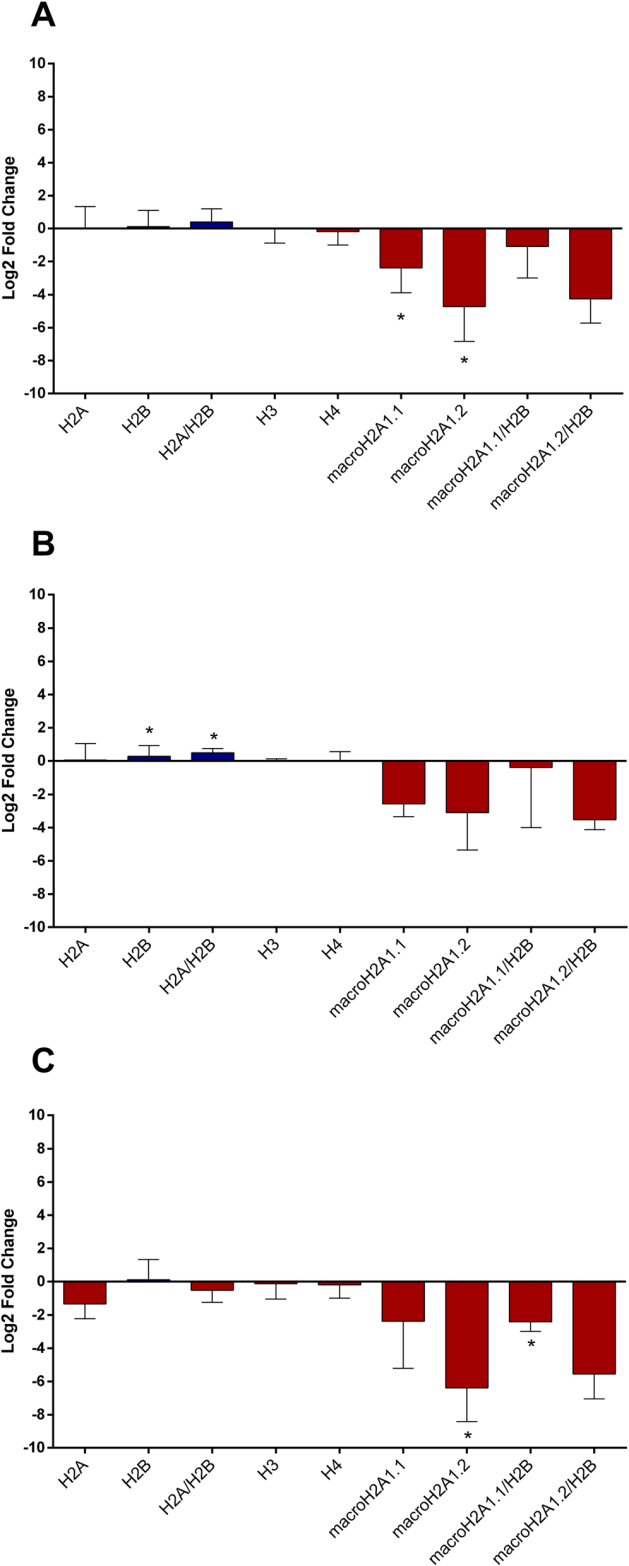
Differences in histone levels between MAFLD patients with steatosis grade 1 (*n* = 9) and those with steatosis 3 (*n* = 9). These groups were matched for age, sex and BMI and each of them was constituted by 4 lean and 5 overweight patients. Results are reported as log^2^ fold change of steatosis grade 3 compared with steatosis grade 1 in lean/overweight (**a**), overweight (**b**) and lean (**c**) patients. *p < 0.05 based on the Student’s *t* test

Upon protocol optimization with a single antibody, we sought to implement a multi-channel flow imaging methodology on ImageStream(X), to verify the possibility of imaging single histone staining on different channels. We took into account the 4 canonical histones (H2A, H2B, H3, H4) and 2 large variants of histone H2A (macroH2A1.1 and macroH2A1.2), and we processed the serum of 9 selected patients with steatosis grade 1 and the serum of 9 patients with steatosis grade 3. In order to perform a comparative analysis, these patients were matched for age, sex and BMI. Thus, no difference in BMI between patients with steatosis grade 1 and those with steatosis grade 3 was evident (median = 25.3, IQR = 23.6–28.0, and median = 24.8; IQR = 23.3–28.3, respectively), and each subgroup consisted of 4 lean and 5 overweight individuals. Using specific primary and secondary antibodies, we were able to image each of the six histones in six different imaging channels (Fig. 5). Early studies have indicated that histone dimers may serve as a stable intermediate in histone assembly, and no trimers form [34]. Each histone type can be seen as an interchangeable subunit of a complex in which the dimer species is the most stable sub-complex [34]. In particular, in vitro, the histones form preferentially H2A-H2B heterodimers and H3-H4 heterotetramers [35]. The H2A/H2B dimer binds onto the H3/H4 tetramer due to interactions between H4 and H2B, in the presence of DNA [35]. For these reasons, we assayed the abovementioned 6 individual histones (H2A, H2B, H3, H4, macroH2A1.1 and macroH2A1.2) together with the following biological dimers: H2A/H2B, macroH2A1.1/H2B, and macroH2A1.2/H2B. Figure 6 shows a representative image of the multi-channel detecting of macroH2A1.2/H2B histone dimer. We report here a significant depletion of the levels of histone variants macroH2A1.1 and macroH2A1.2 in the serum on non-obese (lean+overweight) MAFLD patients, either individually or in complex with H2B (Fig. 7a). The major contributors to these observed differences seemed to be the lean MAFLD individuals, as the same differences were attenuated and resulted not significant in overweight MAFLD individuals (Fig. 7b versus Fig. 7c). In turn, overweight MAFLD differed from lean MAFLD for the significant upregulation of histone H2A and H2A/H2B dimer (Fig. 7b, c). In summary, we identified quantitative differences in circulating histones that are associated to the severity of steatosis in individuals with lean MAFLD, using a rapid and non-invasive ImageStream(X)-based imaging technology.

## Discussion

Non-invasive markers able to diagnose human lean MAFLD, a growing pathology, are currently scarcely characterized or missing. The present pilot study aimed to analyze the role of intact histones and histone complexes—promising “liquid biopsies” released from various dying cells into the blood circulation—as markers of human lean MAFLD. Using ImageStream(X), we identified a specific signature that characterizes in lean MAFLD, consisting of a marked downregulation of histone variants macroH2A1.1 and macroH2A1.2, either alone or forming a natural dimer with H2B. Interestingly, macroH2A1.2 downregulation nearly doubled the one of histone macroH2A1.1 in lean patients with S3 compared to lean patients with S1. Others and we have previously shown that hepatocytes accumulate high levels of macroH2A1.2, and not macroH2A1.1 during MAFLD pathogenesis, and that macroH2A1.2 participates in the latter process [36, 37]; the opposite mechanism seems to occur in the adipose tissue [38, 39]. As obese and lean individuals with MAFLD differ mostly for the amount and location of body adiposity, it is possible that the differences in the circulating levels of macroH2A1.1/macroH2A1.2 reflect the amount of those histones remained “trapped” within hepatocytes and adipocytes, and not released into the bloodstream by these major fat metabolizing cell types when undergoing cell death. Regardless, the tissues of origin of the histone complexes resulting in the circulating signature of lean MAFLD remain unknown. A new identification strategy relies on the assessment of the composition of circulating DNA/histone complexes, based on the fact that DNA/histones physical association biases the identity of DNA fragments in complex with histones. These fragmentation patterns might contain evidence of the epigenetic landscape of the tissue(s) of origin [40, 41].

MacroH2A1.1 and macroH2A1.2 isoforms originate from alternative splicing of macroH2A1. MacroH2A1.1 macrodomain specifically binds nicotinamide adenine dinucleotide (NAD+)-derived metabolites such as ADP-ribose and O-acetyl-ADP-ribose: this binding of ADP-ribose enables macroH2A1.1 to interact with activated PARP1 enzyme [42]. On the other hand, macroH2A1.2 and macroH2A2 isoforms are unable to bind these metabolites [42].

The role of ADP-ribose metabolism in metabolic regulation and lipid accumulation in peripheral tissues has become increasingly appreciated [43]: circulating levels of macroH2A1.1/macroH2A1.2 might reflect hepatic and adipose tissue ADP-ribose metabolism. Early studies in mice showed that macroH2A1 localization is enriched in the bodies of genes functionally clustering in the area of lipid metabolism [44] and artificially manipulating the macroH2A1 isoform expression impinges on cell lipid accumulation [45]. Potential interaction between macroH2A1 isoforms and lipid-metabolism genes involved in lean MAFLD (CETP, PEMT, PNPLA3) [7, 46] remain to be established.

Intact nucleosomes are thought to be released by dying neutrophils or by their neutrophil extracellular traps (NETs), as it was recently confirmed by an automated ImageStream(X)-based pipeline allowing NET quantification in stimulated circulating human neutrophils [47]. Using ELISA assays, we have shown that intact nucleosomes are strong markers of obesity-associated MAFLD [15], but not of lean MAFLD or NASH. A plausible explanation is that the number of neutrophils in MAFLD patients correlates with BMI overweight/obese MAFLD has been reported to be higher compared to lean MAFLD [48, 49]. Hepatic neutrophil infiltration is a hallmark of NASH and is believed to be associated with liver injury and disease progression by generating reactive oxygen species and producing proinflammatory mediators [50]; however, this phenomenon might remain localized and not result in NET-dependent detectable increase in serum nucleosomes.

The strengths of our study rely on (i) the biopsy-proven diagnosis of MAFLD that allowed histological validation of the clinical phenotype and correlations with the histone levels, beyond doubt and (ii) the establishment of a cost-effective, rapid imaging protocol requiring minimal quantities of serum/blood. Our study has also some limitations that include the low sample size, especially for the ImageStream(X)-based analysis. Indeed, the comparison of histone levels was conducted on a limited sample size (i.e. 9 patients per steatosis grade group), which was sufficient to detect a 70% change in histone levels. Therefore, our analysis was not able to detect a small difference between steatosis grade 1 and grade 3 patients. The same comparison stratified by BMI categories should be interpreted with caution even more, and hence, further large-scale studies should be encouraged to confirm our promising results. For the same reason, the limited sample size did not allow us to take into account additional covariates. Although we partially managed this issue by matching the two groups for age, sex, and BMI, the effect of other clinical and personal characteristics should be considered in further research.

In fact, the impact of multi-morbidities, frequently occurring in individuals suffering from obesity and metabolic syndrome, on circulating histone complex composition remains to be ascertained: a larger sample size is needed to determine the nexus between circulating histones, MAFLD in morbidly obese subjects.

In summary, we identified by a new ImageStream(X) methodological approach a circulating histone signature that, if validated in larger and independent cohorts of diverse ethnic background and age, may be useful to diagnose the severity of steatosis in individuals with lean MAFLD. With this approach, circulating histone complexes can be conveniently and comprehensively investigated, thereby offering novel phenotypic and functional insights on unique MAFLD arising in lean individuals.

## Methods

### Study population

A total of 53 non-obese metabolic-associated fatty liver disease (MAFLD) patients were recruited from the outpatient clinic for Liver Disease at the Catholic University of the Sacred Heart of Rome. Body mass index (BMI) was calculated as weight (kg)/height^2^ (m). Waist circumference was measured using standard procedures. Alanine transaminase (ALT), aspartate transaminase (AST), triglycerides, total, high-density lipoprotein (HDL) and low-density lipoprotein (LDL) cholesterol were measured by standard laboratory methods. Fasting glucose and fasting insulin were also evaluated by automated commercial methods. IR was assessed by the homeostatic model assessment (HOMA) [HOMA-IR = (insulin (μIU/ml) × glucose (mmol/l))/22.5)]. A cut-off value of > 2.5 was considered as an index of IR [51].

The scoring of liver biopsies was performed by independent pathologists unaware of patient status. Adult patients with a histologically confirmed diagnosis of MAFLD [33], overweight but not obese, were selected as follows: 21 patients with steatosis grade 1 (0–33%), 24 patients with steatosis grade 2 (34–66%) and 9 patients with steatosis grade 3 (67–100%). All other causes of liver damage, other than lipid accumulation, were excluded. All patients were negative for the hepatitis B and hepatitis C serological markers, had an alcohol consumption level < 30 g/day of ethanol for men and < 20 g/day for women, were not taking any hepatotoxic drugs and showed no evidence of metabolic disease (Wilson’s disease, hereditary hemochromatosis) or autoimmune disease (autoimmune hepatitis, primitive biliary cirrhosis, primitive sclerosing cholangitis). The main exclusion criteria were the presence of liver cirrhosis, diabetes, or previous bariatric surgery. Histological severity of disease was assessed by an expert pathologist according to the NAFLD activity score (NAS), with systematic evaluation of hepatocellular ballooning and lobular inflammation; fibrosis was also staged according to the recommendations of the NAFLD Clinical Research Network. NASH was diagnosed in the presence of steatosis, lobular inflammation, and hepatocellular ballooning [33].

A total of 120 samples from participants of the Kardiovize Brno 2030 without a histologically confirmed diagnosis of NAFLD study were also used. This prospective study was designed to evaluate traditional and novel risk factors for metabolic disorders and cardiovascular diseases among a randomly selected sample of the urban population of Brno, Czech Republic [52, 53]. Selection criteria and study protocols were fully described elsewhere [52, 53]. In this cohort, we previously demonstrated the association of circulating nucleosome levels with obesity [15]. Here, we compared circulating nucleosome levels of MAFLD patients with non-obese or obese controls from the Kardiovize study. Specifically, Kardiovize participants were classified according to BMI and fatty liver index (FLI) as follows: non-obese individuals with FLI < 30 (*n* = 80), obese subjects with FLI < 30 (*n* = 33), and obese patients with FLI ≥ 30 (*n* = 7).

As previously described [14, 54], FLI was calculated from serum triglycerides, BMI, waist circumference and gamma-glutamyl-transferase (GGT) to predict hepatic steatosis in the general population. The optimal cut-off of 30 was able to identify MAFLD patients with a sensitivity and specificity of 80% and 72%, respectively [55].

All blood samples were drawn at 9 am in recruited overnight fasted patients. We followed the standard and robust blood sampling/storage standard operation procedure (SOP) of the UK BioBank [56].

The Ethics Committees of involved institutions approved the study, and all patients provided written informed consent before enrolment.

### Assessment of circulating nucleosomes

Circulating nucleosomes in serum samples were assayed using commercially available ELISA kits (nucleosomes: Cell Death Detection ELISAPLUS, Roche, Mannheim, Germany), according to manufacturer’s instructions.

### ImageStream(X) protocol optimization

To validate the experiment setup, for each sample stained with a single antibody, first, we set up the power of appropriate laser not to detect any saturated pixels. We used the properties “Raw Max Pixel” and feature “Saturation Count” (accessible in the IDEAS® statistical analysis software package (Amnis Corporation, USA)) which reports the number of saturated pixels in an image (Supplemental Figure 1). Pixel intensities are measured on the camera pixels from 0 to 4095 (12 bit) and therefore become saturated and cannot be quantified after 4095. Single color controls were used to calculate a spectral crosstalk matrix that was applied to the image files in order to isolate probed images to single imaging channels. The resulting compensated image files were analyzed using image-based algorithms available in the IDEAS® statistical analysis software package (Amnis Corporation, USA) and analysis of the results was done with the same software. We started from higher voltage to lower. Finding optimal laser power for each single antibody (laser 488 nm–5 mW, laser 561 nm–20 mW, laser 642–20 mW) was used for the multichannel assay.

Sample preparation: for each case, 50-μl serum blood sample was incubated overnight at 4 °C with 4 primary antibodies from each set, in a ratio of 1:50 (the same ratio for each primary antibody). The phosphate buffer (pH 7.4) was used to dilute them, and the antibodies were added from separate solutions, each separately, one after the other. The next day, the sample was incubated for 2 h at room temperature with 4 fluorescent secondary antibodies from each set, in a ratio of 1: 100 (the same ratio for each secondary antibody, for which dilution was used phosphate buffer - pH 7.4 and the antibodies were added from separate solutions, each separately, one after the other), for 2 h at RT. The samples thus prepared were analyzed by imaging cytometry. To confirm the acellular complexes detected by ImageStream(X) as true histone complexes we used, as a positive controls, recombinant human histone H2A protein (Abcam, ab200295, USA), recombinant human histone H4 (Abcam, ab198115, USA), and human native nucleosomes protein (Sigma-Aldrich, 14-1057, Austria). The first positive control, containing recombinant human histone H2A (20 μg/ml), was incubated with primary antibody anti-H2A antibody (Abcam, ab18255, USA) diluted 1/50 in PBS overnight at 4 °C and with secondary antibody anti-mouse IgG H&L-Alexa Fluor® 647 (Abcam, ab150115, USA) diluted 1:1000 for 2 h at RT. The second positive control, containing recombinant human histone H4 (20 μg/ml), was incubated with primary antibody anti-Histone H4 antibody (Abcam, ab31830, USA) diluted 1/50 in PBS overnight at 4 °C and with secondary antibody anti-mouse IgG H&L-Alexa Fluor® 488 (Abcam, ab150113, USA) diluted 1:1000 for 2 h at RT. The third positive control containing human native nucleosomes protein (100 μg/ml) was incubated with primary antibody anti-histone H2A antibody (Abcam, ab18255, USA) diluted 1/50 in PBS and anti-macroH2A1.1 antibody (ActiveMotif, 39871, USA) (or anti-macroH2A1.2, ActiveMotif, 61427, USA) antibody diluted 1/50 in PBS overnight at 4 °C, followed by secondary antibody anti-mouse IgG H&L-Alexa Fluor® 647 (Abcam, ab150115, USA) diluted 1:1000 and anti-rabbit IgG H&L-Alexa Fluor® 488 (Abcam, ab150077, USA) diluted 1:1000 for 2 h at RT. The fourth positive control containing human native nucleosomes protein (100 μg/ml) was incubated with primary antibody anti-Histone H4 antibody (Abcam, ab31830, USA) diluted 1/50 and anti-macroH2A1.1 antibody (ActiveMotif, 39871, USA) (or anti-macroH2A1.2, ActiveMotif, 61427, USA) antibody diluted 1/50 in PBS overnight at 4 °C and with secondary antibody anti-mouse IgG H&L-Alexa Fluor® 488 (Abcam, ab150113, USA) diluted 1:1000 and anti-rabbit IgG H&L-Alexa Fluor® 594 (Abcam, ab150080, USA) for 2 h at RT.

For the measurement, we used multispectral imaging flow cytometer ImageStream MkII (Amnis Corporation). A sample of 10,000 objects was collected using excitation laser 488 nm (5 mW) for Alexa Fluor® 488 and the fluorescence was collected in channel two (505–560 nm), 561 nm (20 mW) for Alexa Fluor® 594 and fluorescence was collected in channel four (595–642 nm) and 642 nm (5 mW) for Alexa Fluor® 647 and fluorescence was collected in channel five (642–745 nm), the bright field image in channel one and the laser scatter image in channel six. To identify the fluorescence-stained objects within all measured objects, gating of the measured populations was applied to discriminate (a) focused objects and (b) objects with fluorescence. To identify stained cells within all measured objects, gating of the measured populations was applied to discriminate (a) focused objects, (b) round single objects (RSC) and (c) objects with fluorescence. The analysis of the results was done with IDEAS® statistical analysis software package (Amnis Corporation, USA). Representative scatter plots, images and fluorescence of multi-channel detecting of histone complexes are shown in Supplemental Figure 2 (macroH2A1.2/H2B), Supplemental Figure 4 (H2A/H2B) and Supplemental Figure 4 (RSC).

### ImageStream(X) detection of histone complexes in the serum of lean MAFLD patients

We used three staining sets; each consisting of four different primary antibodies and four appropriate secondary antibodies. This strategy was due to the fact that the detection of expression of multiple biomarkers in one sample is limited by the parameters of the imaging flow cytometer. Six channels of detection are available in ImageStream(X), but for fluorescence detection, only 4 are available because one channel must be used for bright-field images and one must be used for dark field (SSC).

First staining set. Primary antibodies: anti-macroH2A1.1 (ActiveMotif, 39871, USA), anti-histone H2B (Abcam, Ab134211, USA), anti-histone H4 (Abcam, Ab31830, USA), anti-histone H3 (Abcam, Ab12079, USA). Secondary antibodies: anti-rabbit IgG H&L-Alexa Fluor® 488 (Abcam, Ab150077, USA), anti-chicken IgY H&L-DyLight® 594 (Abcam, Ab96953, USA), anti-mouse IgG H&L-Alexa Fluor® 647 (Abcam, Ab150115, USA); anti- IgG H&L Alexa Fluor® 555 (Abcam, Ab150130, USA).

Second staining set. Primary antibodies: anti-macroH2A1.2 (ActiveMotif, 61427), anti-histone H2B (Abcam, Ab134211, USA), anti-histone H4 (Abcam, Ab31830, USA); anti-histone H3 (Abcam, Ab12079, USA). Secondary antibodies: anti-rabbit IgG H&L-Alexa Fluor® 488 (Abcam, Ab150077, USA), anti-chicken IgY H&L-DyLight® 594 (Abcam, Ab96953, USA), anti-mouse IgG H&L-Alexa Fluor® 647 (Abcam, Ab150115, USA), anti-IgG H&L Alexa Fluor® 555 (Abcam, Ab150130, USA).

Third staining set. Primary antibodies: anti-mH2A (Abcam, Ab18255, USA), anti-histone H2B (Abcam, Ab134211, USA), anti-histone H4 (Abcam, Ab31830, USA), anti-histone H3 (Abcam, Ab12079, USA). Secondary antibodies: anti-rabbit IgG H&L-Alexa Fluor® 488 (Abcam, Ab150077, USA), anti-chicken IgY H&L-DyLight® 594 (Abcam, Ab96953, USA), anti-mouse IgG H&L-Alexa Fluor® 647 (Abcam, Ab150115, USA), anti-goat IgG H&L Alexa Fluor® 555 (Abcam, Ab150130, USA).

The blood samples (serum) from patients were incubated with primary antibodies (added one by one) diluted 1/50 in PBS overnight at 4 °C and with appropriate secondary antibodies (added one by one) diluted 1:1000 for 2 h at RT. For each stained serum sample, a sample of 10,000 objects was collected using excitation laser 488 nm (5 mW) for Alexa Fluor® 488 and fluorescence was collected in channel two (505–560 nm), 561 nm (20 mW) for Alexa Fluor® 555 and DyLight® 594 and fluorescence was collected in channel three (560–595 nm) and channel four (595–642 nm), 642 nm (5 mW) for Alexa Fluor® 647 and fluorescence was collected in channel five (642–745 nm), the bright-field image in channel one and the laser scatter image in channel six.

To identify fluorescence stained objects within all measured objects, gating of the measured populations was applied to discriminate (a) focused objects and (b) objects with fluorescence. To identify stained cells within all measured objects gating of the measured populations was applied to discriminate (a) focused objects, (b) round single objects (RSC), and (c) objects with fluorescence. Single-color controls were used to calculate a spectral crosstalk matrix that was applied to the image files in order to isolate probed images to single imaging channels. The resulting compensated image files were analyzed using image-based algorithms available in the IDEAS® statistical analysis software package (Amnis Corporation, USA) and analysis of the results was done with the same software.

### Statistical analyses

All statistical analyses were conducted using GraphPad Prism (version 6.0, GraphPad Software, USA) or SPSS Statistics software (version 22.0, IBM Corporation, USA). The Kolmogorov-Smirnov test was first used to test the normality of continuous variables before further analyses. Continuous variables underlying a skewed distribution were compared using the Mann-Whitney *U* or Kruskal-Wallis tests. Log-transformed variables with normal distribution were compared using the Student’s *t* test. Categorical variables were compared using the chi-squared test. The comparison of histone levels was carried out on a subsample of patients with histologically confirmed MAFLD. Specifically, 9 patients with steatosis grade 3 were matched to 9 patients with steatosis grade 1 by age, sex and BMI. Assuming *σ* = ½ μ (where *σ* and *μ* were the standard deviation and the mean of histone levels, respectively), this sample size was sufficient to detect a 70% increase/decrease in histone levels with a significance level *α* = 0.05 and a statistical power of 90%. All statistical tests were two-sided, and *p* values < 0.05 were considered statistically significant.

## Supplementary information

**Additional file 1: Supplemental Figure 1.** Examples of 2 different settings of laser power and objects with saturated (A) without saturated (B) pixels.

**Additional file 2: Supplemental Figure 2.** Representative scatter plots and images, fluorescence signal intensity from Alexa Fluor® 488 and Alexa Fluor® 594 of multi-channel detecting of macroH2A1.2/H2B. A. Scatter plots show single positive histones for macroH2A1.2 histone staining (fluorescence from Alexa Fluor® 488, region -+), single positive histones for H2B histone staining (fluorescence from Alexa Fluor® 594, region +-), double positive histones for macroH2A1.2 and H2B (region ++) and unstained objects (region --). B. Representative images of multi-channel detecting of macroH2A1.2/H2B histone. Region -+ shows single macroH2A1.2 histone staining (fluorescence from Alexa Fluor® 488), region +- single H2B histone staining (fluorescence from Alexa Fluor® 594), region ++ - double macroH2A1.2 and H2B staining and unstained objects (region --).

**Additional file 3: Supplemental Figure 3.** Representative scatter plots and images, fluorescence signal intensity from Alexa Fluor® 488 and Alexa Fluor® 594 of multi-channel detecting H2A/H2B histones. A. Scatter plots show single positive histones for H2A histone staining (fluorescence from Alexa Fluor® 488, region -+), single positive histones for H2B histone staining (fluorescence from Alexa Fluor® 594, region +-), double positive histones for H2A and H2B (region ++) and unstained objects (region --). B. Representative images of multi-channel detecting of H2A/H2B histone. Region -+ shows single macroH2A1.2 histone staining (fluorescence from Alexa Fluor® 488), region +- single H2B histone staining (fluorescence from Alexa Fluor® 594), region ++ double H2A and H2B staining and unstained objects (region --).

**Additional file 4: Supplemental Figure 4.** Representative scatter plots, population distribution for round single cells (RSC).

## Data Availability

The datasets used and/or analyzed during the current study are available from the corresponding author on reasonable request.

## Abbreviations

BMI: Body mass index
CEPT: Cholesteryl ester transfer protein
ELISA: Enzyme-linked immunosorbent assay
HOMA-IR: Homeostatic model assessment–insulin resistance
IQR: Interquartile range
MAFLD: Metabolic-associated fatty liver disease
NAD: Nicotinamide adenine dinucleotide
NAFLD: Non-alcoholic fatty liver disease
NAS: NAFLD Activity Score
NASH: Non-alcoholic steatohepatitis
NETs: Neutrophil extracellular traps
PARP1: poly[ADP-ribose] polymerase 1
PEMT: Phosphatidylethanolamine *N*-methyltransferase
PNPLA3: Patatin-like phospholipase domain-containing 3
