# Supplementary figures and images for: Circulating histone signature of human lean metabolic-associated fatty liver disease (MAFLD)
Source: Clin Epigenetics. 2020 Aug 20;12:126. doi: 10.1186/s13148-020-00917-2 (PMC7441674; doi:10.1186/s13148-020-00917-2)

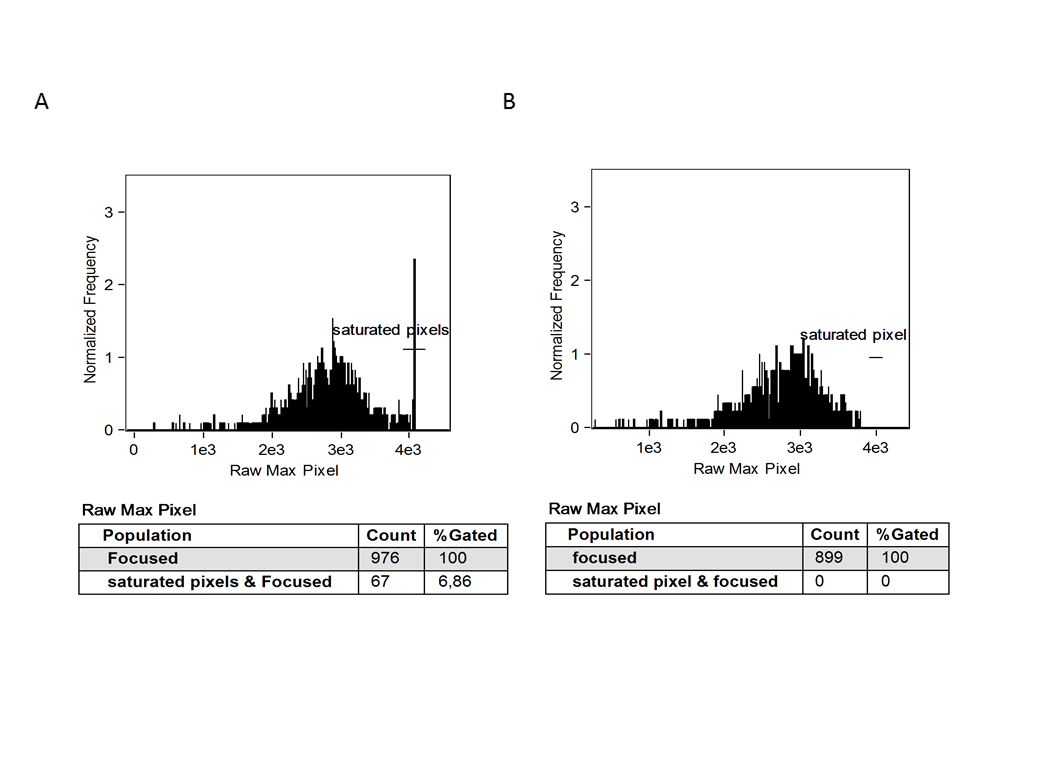

Supplement: Supplementary file 1 — Additional file 1: Supplemental Figure 1. Examples of 2 different settings of laser power and objects with saturated (A) without saturated (B) pixels. [file 13148_2020_917_MOESM1_ESM.tif]

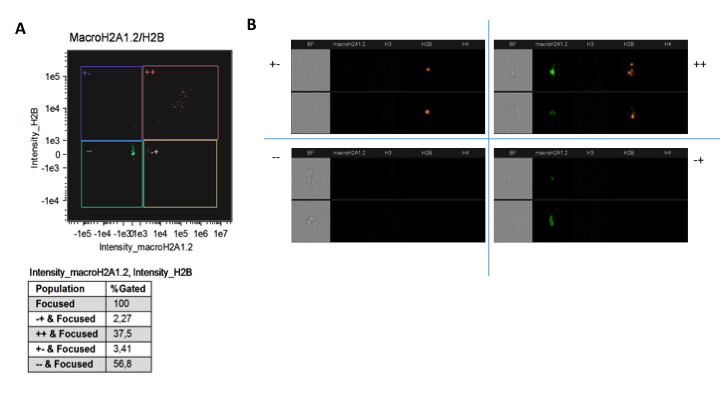

Supplement: Supplementary file 2 — Additional file 2: Supplemental Figure 2. Representative scatter plots and images, fluorescence signal intensity from Alexa Fluor® 488 and Alexa Fluor® 594 of multi-channel detecting of macroH2A1.2/H2B. A. Scatter plots show single positive histones for macroH2A1.2 histone staining (fluorescence from Alexa Fluor® 488, region -+), single positive histones for H2B histone staining (fluorescence from Alexa Fluor® 594, region +-), double positive histones for macroH2A1.2 and H2B (region ++) and unstained objects (region --). B. Representative images of multi-channel detecting of macroH2A1.2/H2B histone. Region -+ shows single macroH2A1.2 histone staining (fluorescence from Alexa Fluor® 488), region +- single H2B histone staining (fluorescence from Alexa Fluor® 594), region ++ - double macroH2A1.2 and H2B staining and unstained objects (region --). [file 13148_2020_917_MOESM2_ESM.tiff]

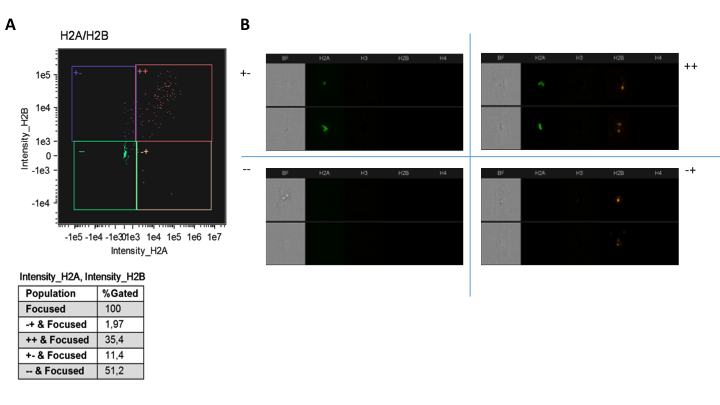

Supplement: Supplementary file 3 — Additional file 3: Supplemental Figure 3. Representative scatter plots and images, fluorescence signal intensity from Alexa Fluor® 488 and Alexa Fluor® 594 of multi-channel detecting H2A/H2B histones. A. Scatter plots show single positive histones for H2A histone staining (fluorescence from Alexa Fluor® 488, region -+), single positive histones for H2B histone staining (fluorescence from Alexa Fluor® 594, region +-), double positive histones for H2A and H2B (region ++) and unstained objects (region --). B. Representative images of multi-channel detecting of H2A/H2B histone. Region -+ shows single macroH2A1.2 histone staining (fluorescence from Alexa Fluor® 488), region +- single H2B histone staining (fluorescence from Alexa Fluor® 594), region ++ double H2A and H2B staining and unstained objects (region --). [file 13148_2020_917_MOESM3_ESM.tiff]

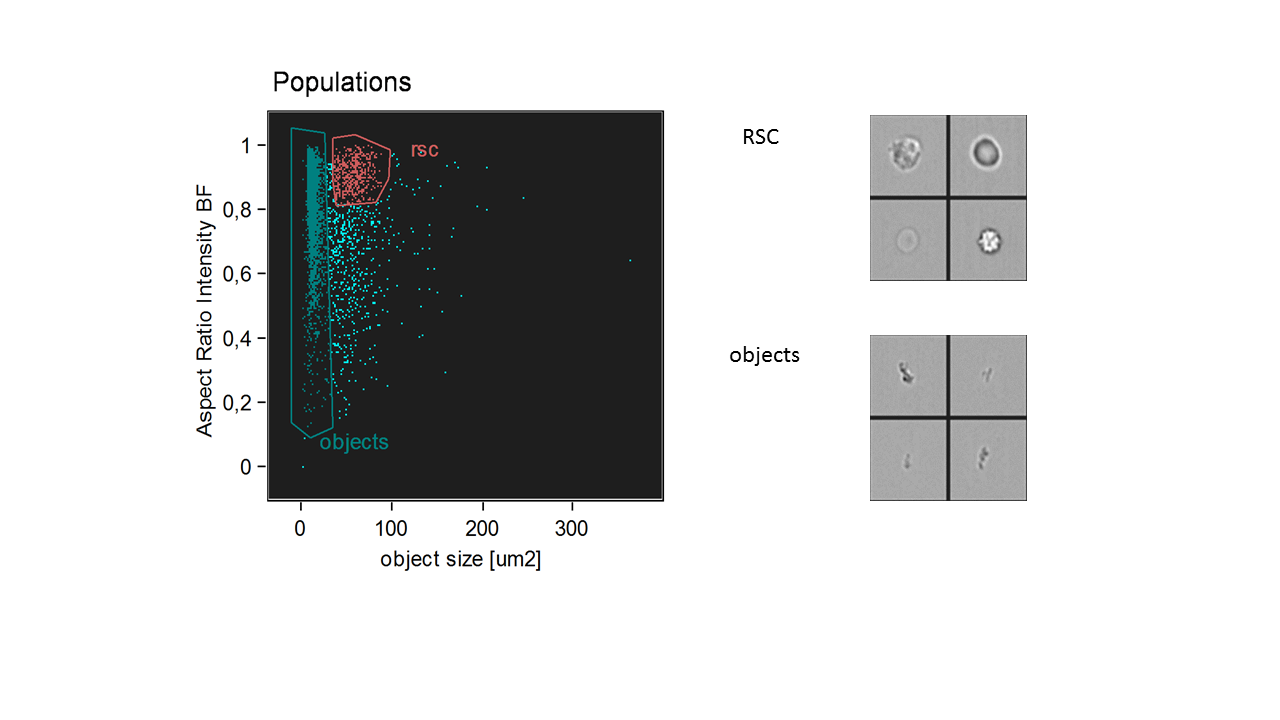

Supplement: Supplementary file 4 — Additional file 4: Supplemental Figure 4. Representative scatter plots, population distribution for round single cells (RSC). [file 13148_2020_917_MOESM4_ESM.tif]
